# Supplementary material for: Hepatitis B virus resistance to tenofovir: fact or fiction? A systematic literature review and structural analysis of drug resistance mechanisms
Source: Wellcome Open Res. 2020 Jun 29;5:151. [Version 1] doi: 10.12688/wellcomeopenres.15992.1 (PMC8033640; doi:10.12688/wellcomeopenres.15992.1)
Supplement: Peer review history

# Peer review: Reviewer response for version 1

**DOI:** 10.21956/wellcomeopenres.17540.r43245

**Published:** 2021-04-14

**Authors:** Suzane K Ono

**Affiliations:** 1 Departamento de Gastroenterologia, Universidade de Sao Paulo, Sao Paulo, Brazil

This is an excellent systematic review on clinical, *in vitro* and *in silico* TNF resistance. The authors reviewed and listed polymorphisms associated to TNF resistance and analyzed the existence of polymorphisms according to HBV genotypes. In addition, compared the polymorphisms in the HBV and HIV RT gene sequence alignments.

 Comments:

 Figure 1. The green box is named within active site? Is it correct? Please verify.

 Figure 1. As mentioned by the authors in the text, polymorphisms at position 80, 173 and 184 are described as compensatory changes, therefore the title “RAMs associated with TDF/TAF …” this could mislead the readers to believe they are all confirmed resistant mutations.

 The urgent need for more HBV sequencing data is important for research and better understanding of the topic; however, it is important to first check patient adherence to treatment (feasible and lower cost) and then look for RAMs. Therefore, I would suggest to change position of item “i” There is urgent need for more HBV sequencing… with item “ii”

Are the rationale for, and objectives of, the Systematic Review clearly stated?

Yes

Is the statistical analysis and its interpretation appropriate?

Not applicable

Are sufficient details of the methods and analysis provided to allow replication by others?

Yes

Are the conclusions drawn adequately supported by the results presented in the review?

Yes

Reviewer Expertise:

Hepatology - hepatitis B.

I confirm that I have read this submission and believe that I have an appropriate level of expertise to confirm that it is of an acceptable scientific standard.

# Peer review: Reviewer response for version 1

**DOI:** 10.21956/wellcomeopenres.17540.r43247

**Published:** 2021-04-08

**Authors:** Azwidowi Lukhwareni

**Affiliations:** 1 Department of Medical Virology, University of Pretoria and National Health Laboratory Service, Tshwane Academic Division, Pretoria, South Africa

TVF Abbreviation was not explained in full.

 This is an excellent systematic review. There is still however limited data regarding HBV primary resistance against TFV or TFV. The data is even sparse in low/middle income settings such as sub Saharan Africa where routine monitoring of HBV viral load on treatment is rarely undertaken despite high usage of TFV as part of HIV/AIDS management. While some resistance mutations have been reported, phenotypic studies are lacking regarding primary TFV resistance mutation. In a recent Gilead HBV clinical studies, all the patients harbouring rtA194T or CYE/CYEI (S106C, H126Y, D134E and L269I) at baseline achieved viral suppression by week 96 after TDF or TAF treatment. No patients developed an rtA194T mutation or > 1 substitution of CYEI, and the number of patients losing any substitutions of CYEI (n = 17) was similar to the number who developed a single substitution of CYEI (n = 32) during treatment. Phenotypic evaluation of the site‐directed mutant panel containing these mutations with or without other resistance mutations did not demonstrate a significant shift in TFV and TAF potency *in vitro*. No evidence of rtA194T and CYEI conferring resistance to TDF or TAF was observed based on the treatment responses to TDF or TAF in patients with mutations at baseline, the lack of selection of mutations after starting TDF or TAF treatment and no change in susceptibility to TFV or TAF in vitro. Overall, rtA194T and CYEI did not appear to confer resistance in any patient on a TDF or TAF regimen.

Are the rationale for, and objectives of, the Systematic Review clearly stated?

Yes

Is the statistical analysis and its interpretation appropriate?

Not applicable

Are sufficient details of the methods and analysis provided to allow replication by others?

Yes

Are the conclusions drawn adequately supported by the results presented in the review?

Yes

Reviewer Expertise:

Infectious diseases.

I confirm that I have read this submission and believe that I have an appropriate level of expertise to confirm that it is of an acceptable scientific standard.

# Peer review: Reviewer response for version 1

**DOI:** 10.21956/wellcomeopenres.17540.r41653

**Published:** 2020-12-16

**Authors:** Dongping Xu

**Affiliations:** 1 Institute of Infectious Diseases, The Fifth Medical Center of Chinese PLA General Hospital (Beijing 302 Hospital), Beijing, China

This is an interesting systematic review. Whether HBV may develop primary resistance to tenofovir (TFV or TDF) remains controversial. Some resistance mutations early reported have been denied by other investigators, such as for rtA194T. Recently, a Korean group reported that a quadruple mutation pattern could confer virus resistant to TFV. However, the results were also questioned for this mutation pattern was also reported being detected in TFV-naive patients. In fact, TFV and adevofir (ADV) had similar structure of nucleotide analogue with similar viral suppression ability. TFV is superior to ADV just due to much lighter side-effect. As a result, the daily dose of TFV is 30 fold higher over the dose of ADV (300 mg for TFV vs. 10 mg for ADV). Therefore, since ADV resistance mutation (rtN236T, rtA181V) which confer at least 2-fold increase of EC50 could lead to resistance, a primary TFV-resistance mutation or mutation pattern should at least confer 60-fold increase of EC50. It is a pity that no convincing data are available to confirm that a mutation or a mutation pattern could certainly confer such an increase of EC50 to TFV. From this view, currently reported HBV mutations are lack of full evidence from phenotypic analysis to be a primary TFV-resistance mutation. For my part, only a few reported mutation(s) such as the quadruple mutation pattern could be termed as TFV-resistance-associated mutations. The mutations could apparently increase EC50 (but less than 60 fold) and thus decrease the effect of TFV. With some other influencing factor, the resistance is likely to occur. In general, this systemic review comprehensively collected and analyzed relevant literatures. The analysis conclusion was adequate with novelty. My only concern is whether it is appropriate to term all mutations listed in Figure 1 as “mutations associated with TFV”, because supportive evidence for most of them to be a TFV-associated mutation is weak or even lack. Probably they should be described as “mutations potentially associated with TFV”.

Are the rationale for, and objectives of, the Systematic Review clearly stated?

Yes

Is the statistical analysis and its interpretation appropriate?

Not applicable

Are sufficient details of the methods and analysis provided to allow replication by others?

Yes

Are the conclusions drawn adequately supported by the results presented in the review?

Yes

Reviewer Expertise:

Infectious Diseases.

I confirm that I have read this submission and believe that I have an appropriate level of expertise to confirm that it is of an acceptable scientific standard.
